# Risk factors for reoperation after lumbar spine surgery in a 10-year Korean national health insurance service health examinee cohort

**DOI:** 10.1038/s41598-022-08376-w

**Published:** 2022-03-17

**Authors:** Sung Hyun Noh, Pyung Goo Cho, Keung Nyun Kim, Boeun Lee, Jae Kwang Lee, Sang Hyun Kim

**Affiliations:** 1 Department of Neurosurgery, Ajou University School of Medicine, Suwon, Republic of Korea; 2 Department of Neurosurgery, Yonsei University College of Medicine, Seoul, Korea; 3 Department of Neurosurgery, Spine and Spinal Cord Institute, Severance Hospital, Yonsei University College of Medicine, Seoul, Korea; 4 Department of Neurosurgery, National Health Insurance Service Ilsan Hospital, Goyang, Korea; 5 Research Institute, National Health Insurance Service Ilsan Hospital, Goyang, Korea

**Keywords:** Neurological disorders, Diseases, Health care, Medical research, Risk factors

## Abstract

Degenerative lumbar spine disease is becoming increasingly prevalent in the aging population. Surgical treatment is the standard treatment modality for intractable cases, but the reoperation rate remains high. We conducted this study to longitudinally evaluate the impact of health risk factors on the risk of lumbar spine reoperation in Koreans aged over 40 years. Subjects aged > 40 years who underwent their first lumbar spinal surgery between January 2005 and December 2008 were selected and followed up until 2015. A total of 6300 people were included. The reoperation rate during the 10-year follow-up period was 13.2% (831/6300 patients). The reoperation rate was the highest in patients in their 60 s (15.4%, *P* < 0.05). The reoperation rates were also significantly higher in men (vs. women: 14.7% vs. 11.7%, *P* < 0.05), smokers (vs. non-smokers: 15.2% vs. 12.7%, *P* < 0.05), alcohol drinkers (vs. non-drinkers: 14.7% vs. 12.4%, *P* < 0.05), and those with a higher Charlson Comorbidity Index (CCI) score (CCI 0, 11.6%; 1–2, 13.2%; and ≥ 3, 15%; *P* < 0.05). Among patients undergoing lumbar spine surgery, reoperation is performed in 13.2% of patients within 10 years. Male sex, age in the 60 s, alcohol use, smoking, higher Hgb and a high CCI score increased the risk of reoperation after lumbar spine operation.

## Introduction

Degenerative lumbar spine disease is a common aging-related spinal disorder; its incidence is increasing in the current aging society^1^. Surgical treatment is established as the standard treatment modality for intractable cases^2^. The rate of lumbar spine surgery has increased by more than twofold owing to not only an increase in the prevalence of degenerative lumbar spine disease but also to improvements in surgical techniques, favorable outcomes, and an increase in the number of hospitals and surgeons^3,4^. However, some patients require reoperation because of complications including infection, fusion failure, and persistent pain and diseases related to progressive degenerative changes or an unrelated previous surgery^5,6^. Despite improvements in surgical skills and techniques, the reoperation rate remains unimproved, with a 10-year reoperation rate of approximately 20%^7^. Considering the high prevalence and chronicity of degenerative lumbar spine disease, it is important to understand the risk factors affecting reoperation^1^.

Population-based studies have shown the longitudinal trends in the lumbar reoperation rate and its relationships with coexisting diseases, demographic characteristics, primary operation type, and preoperative spinal pathology^8^. However, research on the influence of lifestyle-related factors including smoking, drinking, and exercise on the risk of reoperation after lumbar surgery is lacking. The Korean National Health Insurance Service (NHIS) covers the health insurance of approximately 96% of Koreans aged > 40 years^9^. All insured persons and their dependents are encouraged to participate in a periodic, mostly biennial, general health examination. Data from these examinations are then periodically collected by the NHIS-Health Screening Cohort (NHIS-HEALS) to obtain a large dataset on factors such as smoking, drinking, height, weight, blood pressure, and basic biochemical data^9^. The present study aimed to longitudinally evaluate the impact of health risk factors on lumbar spine reoperation among Koreans aged over 40 years.

## Methods

### Study design and data source

This retrospective study was approved by the Institutional Review Board of the hospital (Ajou University Institutional Review Board and Ethics Committee 2021-01-009). All methods were performed in accordance with the relevant guidelines and regulations. All participants agreed to fill out an informed consent form. The Korean NHIS database was used to create cohorts of all Korean health examinees who underwent the biennial health examination. The NHIS, a single insurance company, was started in 2006 by consolidating more than 366 medical insurance organizations for efficient system operation in Korea^10^.

All insured individuals and their dependents are classified as insured employees, insured self-employed individuals, or medical aid beneficiaries. They are encouraged to participate in a general health examination, usually conducted every other year^9^. The NHIS-HEALS database includes demographic and medical treatment data as well as information on health risk factors such as smoking, drinking, height, weight, blood pressure, fasting blood glucose levels, and exercise^9^.

### Patient selection

**Figure 1:**
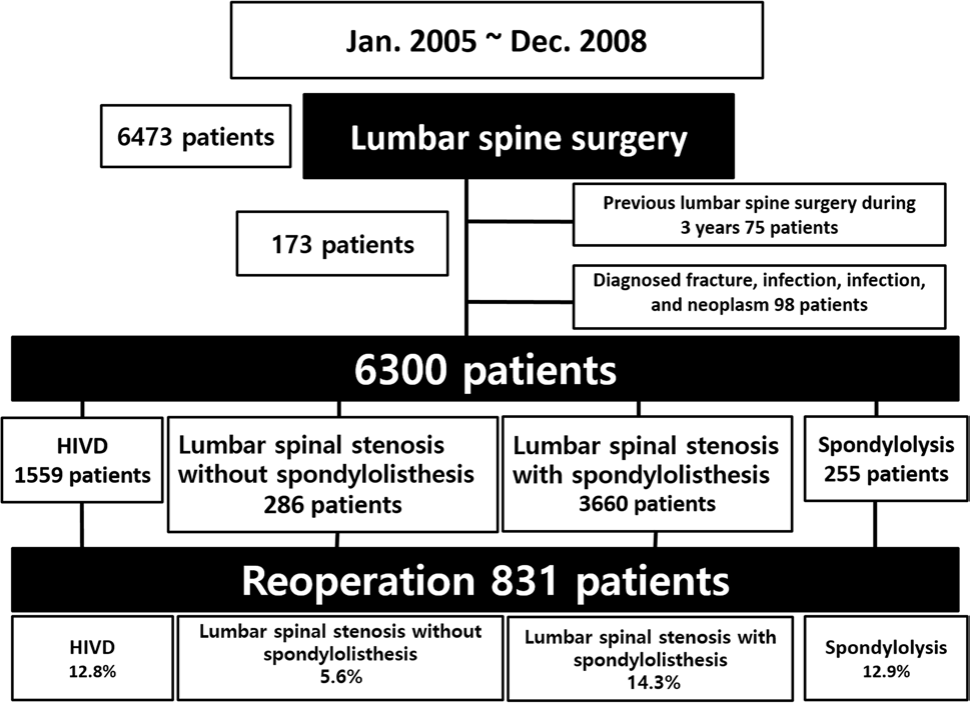
Cohort definition. Individuals aged > 40 years who underwent their first lumbar spinal surgery between January 2005 and December 2008 were selected and followed-up until 2015. Among the 6473 patients identified, 173 patients who underwent the surgery in the previous 3 years (2002–2004, *n* = 75) and who had undergone lumbar spinal surgery for fractures, infections, tumors, or inflammatory disease (*n* = 98) were excluded. Therefore, a total of 6300 patients were enrolled, and 831 of them underwent revision surgery.

Individuals aged > 40 years who underwent their first lumbar spinal surgery between January 2005 and December 2008 were selected and followed-up until 2015. The inclusion criteria were patients who underwent open discectomy (OD), decompressive laminectomy, percutaneous endoscopic lumbar discectomy (PELD), and spinal fusion to treat degenerative lumbar disease. The exclusion criteria were patients who underwent lumbar spine surgery in the previous 3 years (2002–2004) and lumbar spine surgery for fractures, infections, tumors, or inflammatory disease. Among the 6473 patients identified, 173 patients who underwent the surgery in the previous 3 years (2002–2004, *n* = 75) and who had undergone lumbar spinal surgery for fractures, infections, tumors, or inflammatory disease (*n* = 98) were excluded. Finally, a total of 6300 people were enrolled, and 831 of them underwent revision surgery. The patient selection flowchart is shown in Fig. 1.

### Variables

The operation codes were standardized to file claims for medical fees to the NHIS. Lumbar spine-related surgery included OD, laminectomy, PELD, and spinal fusion. The specific surgical level was not evaluated as the claims data did not specify the detailed surgical level. Thus, reoperation in this study was presumed to include surgery at both the index level and another lumbar level^6^. Reoperation was defined as lumbar surgery in the follow-up period after a diagnosis of lumbar degenerative disease. Variables were identified using the International Classification of Diseases, Tenth Revision (ICD-10) codes. Lumbar spine diseases included lumbar disc herniation (codes: M4720-9, M5410, M5412, M5413, M5419, and M511), lumbar spinal stenosis without spondylolisthesis (codes: M4800 and M4805-8), lumbar spinal stenosis with spondylolisthesis (codes: M431 and M4315), and lumbar spondylolysis (codes: M430 and M4306-9). Lumbar spine surgery included OD (code N1493), which contains medial lamino-facetectomy, biportal endoscopic discectomy, and biportal endoscopic decompression; laminectomy (codes: N1499 and N2499), PELD (code N1494); and spinal fusion (codes: N0466, N1466, N0469, N2470, N1460, and N1469).

Comorbidities were evaluated using the modified Charlson Comorbidity Index (CCI) presented by Quan et al.^11^ Coexisting disease was defined as three or more visits to an outpatient clinic or at least 2 days of hospitalization with a diagnosis specified following the Major Disease Code of the year of enrollment^12^. Age was divided into five groups: 40 s, 50 s, 60 s, 70 s, and ≥ 80 s. Residence was divided into 16 categories based on the registration address. Eligibility for health insurance was classified into six categories: 1, regional member household head; 2, regional member household member; 5, employee; 6, dependent of an employee; 7, household head; and 8, household member.

Insurance premiums were divided into 11 levels according to income, with the level increasing with increasing income. Participants were categorized according to smoking status as non-smoker (no smoking or a history of smoking with cessation) and current (active smoking). Alcohol use was categorized as no (do not drink) and yes (drink 2–3 times a month, 1–2 times a week, 3–4 times a week, and every day). The frequency of exercise was divided into three categories: 1 (no exercise), 2 (≥ 30 min, 1–2 times a week or 3–4 times a week), and 3 (≥ 30 min daily or 5–6 times a week).

### Statistical analysis

Patient characteristics were presented as the mean ± standard deviation for continuous variables and the frequency (percentage) for categorical variables. The cumulative incidence and 95% confidence interval (95% CI) of reoperation were calculated using a nonparametric method. Pearson’s chi-square test was performed to investigate differences between the reoperation group and non-reoperation group. The significant influencing factors of reoperation were identified according to hazard ratios and 95% CIs obtained via univariate and multivariate Cox proportional hazard regression analyses. The covariates used for multivariate are sex, age, BMI, smoking, alcohol drinking, exercise, blood sugar, total cholesterol, hemoglobin, and CCI. All statistical analyses were performed using SPSS (version 23.0, SPSS, Chicago, IL, USA) and SAS (version 9.2, SAS, Cary, NC, USA). A *P*-value < 0.05 was considered statistically significant.

## Results

### Patient characteristics

A total of 6300 patients (3076 [49%] men and 3224 [51%] women) with a mean age of 60.5 ± 9.1 years were evaluated. The patients’ clinicodemographic characteristics are described in Table 1. Most of the patients were in their 60 s, followed by those in their 50 s, 70 s, and 40 s, with the minority being in their 80 s. Lumbar spinal stenosis with spondylolisthesis (61%) was the most common diagnosis, followed by lumbar disc herniation (28%), lumbar spinal stenosis without spondylolisthesis (7%), and lumbar spondylolysis (4%). The most common surgical approach was OD (43%), followed by spinal fusion (39%), laminectomy (14%), and PELD (4%).

**Table 1:** Patient demographics.

Category	Number
**Sex**	
Male	**3076 (49%)**
Female	**3224 (51%)**
**Diagnosis**	
Lumbar disc herniation	**1739 (28%)**
Lumbar spinal stenosis without spondylolisthesis	**466 (7%)**
Lumbar spinal stenosis with spondylolisthesis	**3840 (61%)**
Lumbar spondylolysis	**255 (4%)**
**Surgical method**	
Open discectomy	**2696 (43%)**
PELD	**242 (4%)**
Fusion	**2433 (39%)**
Laminectomy	**929 (14%)**
**Age, years**	
40 ~ 49	**908 (14%)**
50 ~ 59	**2007 (32%)**
60 ~ 69	**2272 (36%)**
70 ~ 79	**1047 (17%)**
80 ~	**66 (1%)**
**Residence**	
Seoul	**940 (15%)**
Pusan	**356 (6%)**
Daegu	**248 (4%)**
Incheon	**330 (4%)**
Gwangju	**204 (3%)**
Daejeon	**224 (4%)**
Ulsan	**161 (3%)**
Gyeonggi-do	**1212 (19%)**
Gangwon-do	**244 (4%)**
Chungbuk-do	**237 (4%)**
Chungnam-do	**420 (7%)**
Jeonbuk-do	**371 (6%)**
Jeonnam-do	**420 (7%)**
Gyeongbuk-do	**441 (7%)**
Gyeongnam-do	**433 (6%)**
Jeju-do	**59 (1%)**
**Insuracne eligibility**	
1	**1254 (19%)**
2	**861 (17%)**
5	**1211 (18%)**
6	**2834 (44%)**
7	**111 (1.5%)**
8	**29 (0.5%)**
**Health insurance premium**	
Medical benefit	**140 (2%)**
1	**392 (6%)**
2	**365 (6%)**
3	**384 (6%)**
4	**479 (8%)**
5	**480 (8%)**
6	**625 (10%)**
7	**645 (10%)**
8	**727 (11%)**
9	**981 (15%)**
10	**1082 (18%)**
**BMI, kg/m**^**2**^	
< 20	**345 (5%)**
20 < < 25	**3320 (52%)**
25 <	**2635 (43%)**
**Smoking**	
Yes	**1158 (18%)**
No	**5142 (82%)**
**Alcohol drinking**	
Yes	**2194 (35%)**
No	**4106 (65%)**
**Exercise**	
1	**3737 (59%)**
2	**1846 (29%)**
3	**717 (12%)**
**CCI**	
0	**1650 (26%)**
1–2	**3258 (51%)**
≥ 3	**1392 (23%)**
**Blood sugar (mg/dl)**	**100.49 ± 30.43**
**Total Cholesterol (mg/dl)**	**202.15 ± 38.14**
**Hemoglobin (g)**	**13.78 ± 1.44**

PELD, Percutaneous endoscopic lumbar discectomy; BMI, Body mass index;

CCI, Charlson comorbidity index.

### Comparisons between the no reoperation and reoperation groups

Reoperation was needed in 831 of the 6300 patients (13.2%). The overall 1-, 2-, 3-, 5-, and 11-year cumulative incidence rates of reoperation were 3.2%, 4.8%, 6.3%, 9.1%, and 13.2%, respectively. Table 2 shows the patient characteristics according to the occurrence of reoperation. In total, 453 of the 3076 male patients (14.7%) and 376 of the 3224 female patients (11.7%) underwent reoperation, with the rate being significantly higher in men than in women (*P* = 0.0004). Meanwhile, although the reoperation rates were higher, the difference did not reach significance in those with lumbar spinal stenosis with spondylolisthesis (14.3%) and those who underwent PELD as the first surgery (22.3%). However, the reoperation rates were significantly higher in the 60 s group than in the other age groups (15.5%, *P* = 0.0022), in smokers than in non-smokers (15.2% vs 12.7%, *P* = 0.0254), in alcohol drinkers than in non-alcohol drinkers (14.7% vs 12.4%, *P* = 0.0086), and in those with a high CCI score than in those with a low CCI score (CCI 0: 11.6%; CCI 1–2, 13.2%; CCI ≥ 3, 15%; *P* = 0.0202). The other factors did not show significant difference.

**Table 2:** Comparisons between the No reoperation and reoperation groups.

Category	No reoperation (*n* = 5469)	Reoperation (*n* = 831)	*P*-value
Sex			
Male	2623	453 (14.7%)	
Female	2846	378 (11.7%)	0.0004*
Diagnosis			
Lumbar disc herniation	1516	223 (12.8%)	
Lumbar spinal stenosis without spondylolisthesis	440	26 (5.6%)	
Lumbar spinal stenosis with spondylolisthesis	3291	549 (14.3%)	
Lumbar spondylolysis	222	33 (12.9%)	0.5272
Surgical method			
Open discectomy	2323	373 (13.8%)	
PELD	188	54 (22.3%)	
Fusion	2163	270 (11.1%)	
Laminectomy	795	134 (14.4%)	0.4521
Age, years			
40 ~ 49	804	104 (11.5%)	
50 ~ 59	1771	236 (11.8%)	
60 ~ 69	1921	351 (15.5%)	
70 ~ 79	914	133 (12.7%)	
80 ~	59	7 (10.6%)	0.0022*
Residence			
Seoul	812	128	
Pusan	312	44	
Daegu	218	30	
Incheon	292	38	
Gwangju	179	25	
Daejeon	187	37	
Ulsan	143	18	
Gyeonggi-do	1053	159	
Gangwon-do	204	40	
Chungbuk-do	204	33	
Chungnam-do	369	51	
Jeonbuk-do	312	59	
Jeonnam-do	377	43	
Gyeongbuk-do	380	61	
Gyeongnam-do	372	61	
Jeju-do	55	4	0.4379
Insuracne eligibility			
1	1078	176	
2	757	104	
5	1047	164	
6	2466	368	
7	94	17	
8	27	2	0.6406
Health insurance premium			
Medical benefit	121	19	
1	344	48	
2	314	51	
3	340	44	
4	412	67	
5	422	58	
6	540	85	
7	548	97	
8	625	102	
9	857	124	
10	946	136	0.8635
BMI, kg/m^2^			
< 20	306	39 (11.3%)	
20 < < 25	2892	428 (12.9%)	
25 <	2271	364 (13.8%)	0.3286
Smoking			
Yes	982	176	
No	4487	655	0.0254*
Alcohol drinking			
Yes	1871	323	
No	3598	508	0.0086*
Exercise			
1	3250	487	
2	1616	230	
3	603	114	0.0627
CCI			
0	1459	191	
1–2	2827	431	
≥ 3	1183	209	0.0202*
Blood sugar	100.57 ± 30.60	99.96 ± 29.29	0.5929
Total cholesterol	202.29 ± 38.42	201.27 ± 36.27	0.4569
Hemoglobin	13.76 ± 1.44	13.90 ± 1.42	0.086

PELD, Percutaneous endoscopic lumbar discectomy; BMI, Body mass index;

CCI, Charlson comorbidity index.

*Statistically significant.

### Cumulative number and incidence of reoperation

Table 3 shows the cumulative number and incidence of reoperation. The overall cumulative incidence of reoperation was 3.2% at 1 year, 4.8% at 2 years, 6.3% at 3 years, 9.1% at 5 years, and 13.2% at 11 years.

**Table 3:** Cumulative number and incidence of reoperation.

	OD	Lami	PELD	Fusion	Total
3 month	47	11	10	10	78
6 month	78	15	12	23	128
1 year	117	31	16	39	203 (3.2%)
2 years	161	54	22	67	304 (4.8%)
3 years	199	67	33	98	397 (6.3%)
4 years	234	88	37	126	485 (7.7%)
5 years	272	102	40	162	576 (9.1%)
6 years	310	111	45	191	657 (10.4%)
7 years	334	124	47	221	726 (11.5%)
8 years	352	127	50	243	772 (12.3%)
9 years	368	131	51	259	809 (12.8%)
10 years	373	134	54	270	831 (13.2%)

OD, Open discectomy; Lami, Laminectomy; PELD, Percutaneous endoscopic lumbar discectomy.

### Univariate and multivariate logistic regression analyses of factors influencing reoperation

The results of univariate and multivariate logistic regression analyses for the influencing factors of reoperation are shown in Table 4. Univariate logistic regression analysis identified hemoglobin count (*P* = 0.0251) and a CCI score of ≥ 3 as significant factors (*P* = 0.0188). However, only a CCI score of ≥ 3 was a significant factor in multivariate logistic regression analysis (*P* = 0.0208).

**Table 4:** Univariate and multivariate logistic regression analyses of factors influencing reoperation.

Variable		Univariable analysis			Multivariable analysis		
		HR	95% CI	*P*-value	HR	95% CI	*P*-value
Sex	Female	1	−	−	1	−	−
	Male	1.191	0.908–1.561	0.2064	0.978	0.658–1.453	0.911
Age	40 ~ 49	1	−	−	1	−	−
	50 ~ 59	1.109	0.798–1.542	0.5365	1.084	0.776–1.515	0.6349
	60 ~ 69	1.251	0.868–1.803	0.2301	1.191	0.814–1.742	0.3672
	70 ~ 79	1.034	0.595–1.8	0.9045	1.017	0.573–1.805	0.9544
	80 ~	1.322	0.183–9.548	0.7818	1.477	0.199–10.951	0.7031
BMI	< 20	1	−	−	1	−	−
	20 < < 25	1.272	0.667–2.424	0.4657	1.231	0.641–2.365	0.5332
	25 <	1.448	0.755–2.778	0.2652	1.365	0.702–2.654	0.3594
Smoking	No	1	−	−	1	−	−
	Yes	1.204	0.893–1.624	0.2241	1.173	0.836–1.646	0.3553
Alcohol drinking	No	1	−	−	1	−	−
	Yes	1.099	0.844–1.43	0.4837	1.044	0.759–1.435	0.7918
Exercise	1	1	−	−	1	−	−
	2	0.79	0.587–1.062	0.1187	0.753	0.556–1.02	0.0674
	3	1.051	0.694–1.591	0.8142	1.023	0.673–1.554	0.9164
Blood sugar (mg/dl)		1.002	0.997–1.006	0.4223	1	0.995–1.005	0.9936
Total Cholesterol (mg/dl)		1	0.997–1.004	0.9437	1	0.996–1.003	0.8421
Hemoglobin (g)		1.11	1.013–1.216	0.0251*	1.12	0.993–1.262	0.0647
CCI	0	1	−	−	1	−	−
	1 ~ 2	1.243	0.912–1.692	0.1681	1.253	0.915–1.716	0.159
	≥ 3	1.616	1.083–2.412	0.0188*	1.635	1.078–2.481	0.0208*

HR, hazard ratio; CI, confidence interval, PELD, Percutaneous endoscopic lumbar discectomy; BMI, Body mass index; CCI, Charlson comorbidity index.

*Statistically significant.

## Discussion

Data on the influence of lifestyle-related factors, including smoking, drinking, and exercise, on the risk of reoperation after lumbar surgery are limited. This study found that male sex, older age, alcohol use, smoking, and a high CCI score increased the risk of reoperation. To our best knowledge, this study is the first to longitudinally evaluate the impact of lifestyle habits and comorbidities on the risk of reoperation after spine surgery.

There are several studies on reoperation after the first spine surgery^4,6,7,13–15^. Martin et al. assessed the rate of reoperation after decompression or fusion lumbar surgery using data of 26,675 patients registered in the Washington Administration database^7^. The cumulative incidence of reoperation was 19.0% over an 11-year follow-up period, and the reoperation rates did not differ according to diagnosis. Davis et al. reported that the average rate of reoperation after lumbar disc surgery was 6% over 10.8 years^16^. Vik et al.^17^ reported a reoperation rate of 24% over an 8-year follow-up period. Kim et al. showed an increasing trend in the cumulative reoperation rate after spine surgery in Korea in 2008, with the rate being 5.4% at 3 months, 7.4% at 1 year, 9% at 2 years, 10.5% at 3 years, 12.1% at 4 years, and 13.4% at 5 years^4,6^. In their consequent 10-year follow-up study, the overall cumulative incidence of reoperation after lumbar disc surgery was 4% at 1 year, 6% at 2 years, 8% at 3 years, 11% at 5 years, and 16% at 10 years^18^. Jung et al. similarly reported an increasing trend in the overall cumulative incidence of reoperation for spinal canal stenosis, with the rate being 3.7% at 1 year, 6.2% at 2 years, 8.3% at 3 years, 10.8% at 5 years, and 18.4% at 10 years^19^. In line with these findings, the overall cumulative incidence of reoperation in our study was 3.2% at 1 year, 4.8% at 2 years, 6.3% at 3 years, 9.1% at 5 years, and 13.2% at 11 years. Due to the use of NHIS-HEALS data, the reoperation rate was similar to those in other previous studies.

Several studies have proposed male sex as a risk factor for reoperation. Park et al.^8^ and Kim et al.^18^ reported higher rates of reoperation in men than in women. This could be because back pain is more common in male patients than in female patients^20^. Further, preoperative low back pain tends to be associated with worse surgical outcomes in patients with spinal disorders^21^. Consistently, we found significantly higher rates of reoperation in men than in women (14.7% vs 11.7%, *P* = 0.0004).

Several studies have published the reoperation rate according to the method of spine surgery. Martin et al.^7^ compared the rate of reoperation between fusion only and decompression only and found higher rates with the former approach (16.7% vs 15.8%). Kim et al. compared the 90-day reoperation rates among fusion, laminectomy, OD, endoscopic discectomy, and nucleolysis and found higher rates with laminectomy and lower rates with endoscopic discectomy than with OD. Meanwhile, there was no significant difference in the reoperation rate between the other surgical methods. Kim et al.^18^ also found a higher reoperation rate with PELD than with fusion, OD, and laminectomy. In the current study, the reoperation rate with PELD was 22.3%; laminectomy, 14.4%; OD, 13.8%; and fusion, 11.1%, with no significant differences.

Age is also proposed as a risk factor for reoperation. Kim et al.^6^ reported that age increases the risk of reoperation because of aging-related degenerative changes. In general, the reason for reoperation after spinal surgery is thought to be recurrence at the site of disc surgery or adjacent segment disease at the upper and lower levels or nonunion. Martin et al. compared the reoperation rates between patients aged ≤ 60 years and > 60 years and found significantly higher rates in those aged > 60 years^7^. Similarly, the rate of reoperation in our study was significantly higher in the 60 s group than in the other age groups. (*P* = 0.0022).

Alcohol use and smoking have been reported to influence the outcome of spinal reoperation^22–25^. In the study by Anderson et al.^22^, 51% and 33% of patients with and without recurrent lumbar disc herniation (LDH) were smokers, respectively. Importantly, smoking had a significant effect on the recurrence of LDH (*P* < 0.05). Fritzell et al.^25^ also reported that smoking is a risk factor for no improvement in leg pain after the first surgery. Passias et al.^23^ found that alcohol consumption more than two times a week was a risk factor for pseudoarthrosis after spine surgery and eventually led to reoperation. In contrast, Elsamadicy et al.^24^ reported that alcohol use had no effect on the 30-day readmission or complication rates after adult spinal deformity surgery. In our study, alcohol consumption and smoking were significant risk factors for reoperation. (*P* = 0.0086/*P* = 0.0254).

The associations between comorbid diseases and the risk of reoperation after spinal surgery have been investigated in several studies^4,6,8,19^. Kim et al.^6^ reported that the presence of comorbidities increased the risk of reoperation within 90 days of spinal surgery. Park et al.^8^ also showed that comorbid diabetes was an important risk factor for reoperation. Jung et al.^19^ reported a higher reoperation rate in patients with a CCI score of 1–2 than in patients with a CCI score of 0. In our study, there was a trend toward a significantly higher rate of reoperation with increasing CCI scores (*P* = 0.0202). A multivariate analysis revealed that CCI score ≥ 3 was the only risk factor for reoperation (*P* = 0.0208).

As with other nationwide big data studies, our research has some limitations. First, we used the NHIS-HEALS cohort and not the entire population. Second, clinical information about pain levels, neurological condition, quality of life, functional outcomes, radiographic findings, the complexity of the operation, and the reasons for reoperation was not available. Third, analyzing the rate of reoperation can underestimate or overestimate the rate of surgical failure. Fourth, reoperation included secondary spinal surgery that was not specifically performed at the index level. Fifth, this was not a randomized comparative study; the choice of surgical method might have varied according to surgeon and facility. Sixth, the findings from this patient population from a national database may not be generalizable to an international population. However, unlike the nationwide cohorts and sample cohorts used in previous big data studies, the NHIS-HEALS cohort includes information on lifestyle factors and certain blood parameters. This study identified further risk factors associated with lumbar spine reoperation.

In conclusion, we found that among Korean patients aged > 40 years who undergo lumbar spine surgery, 13.2% undergo reoperation within 11 years. Male sex, age in the 60 s, alcohol use, smoking, higher Hgb, and a high CCI score increased the risk of reoperation. This information will be beneficial for lowering reoperation rates in these patients.

## References

[1] Tian W, Lv Y, Liu Y, Xiao B, Han X (2014). The high prevalence of symptomatic degenerative lumbar osteoarthritis in Chinese adults. Spine.

[2] Weinstein JN (2007). Surgical versus nonsurgical treatment for lumbar degenerative spondylolisthesis. N. Engl. J. Med..

[3] Kim, C., et al. Increased proportion of fusion surgery for degenerative lumbar spondylolisthesis and changes in reoperation rate: a nationwide cohort study with a minimum 5-year follow-up. *Spine (Phila Pa. 1976)*. **44**, 346–354 (2019). 10.1097/BRS.0000000000002805 30028778

[4] Kim CH (2018). Increased volume of surgery for lumbar spinal stenosis and changes in surgical methods and outcomes: a nationwide cohort study with a 5-year follow-up. World Neurosurg..

[5] Deyo RA, Martin BI, Kreuter W, Jarvik JG, Angier H, Mirza SK (2011). Revision surgery following operations for lumbar stenosis. J. Bone Joint Surg. Am..

[6] Kim, C.H., Chung, C.K., Park, C.S., Choi, B., Kim, M.J., Park, B.J. Reoperation rate after surgery for lumbar herniated intervertebral disc disease: nationwide cohort study. *Spine (Phila Pa. 1976).* **38**, 581–590 (2013). 10.1097/BRS.0b013e318274f9a7 23023591

[7] Martin, B.I., Mirza, S.K., Comstock, B.A., Gray, D.T., Kreuter, W., Deyo, R.A. Reoperation rates following lumbar spine surgery and the influence of spinal fusion procedures. *Spine (Phila Pa. 1976).* **32**, 382–387 (2007). 10.1097/01.brs.0000254104.55716.46 17268274

[8] Park MS (2019). Reoperation rates after posterior lumbar spinal fusion surgery according to preoperative diagnoses: a national population-based cohort study. Clin. Neurol. Neurosurg..

[9] Kim M, Ko M, Han J (2010). Alcohol consumption and mortality from all-cause and cancers among 1.34 million Koreans: the results from the Korea national health insurance corporation's health examinee cohort in 2000. Cancer Causes Control.

[10] Lee J, Lee JS, Park S, Shin SA, Kim K (2017). Cohort profile: the national health insurance service-national sample cohort (NHIS-NSC). South Korea. Int. J. Epidemiol..

[11] Charlson ME, Pompei P, Ales KL, MacKenzie CR (1987). A new method of classifying prognostic comorbidity in longitudinal studies: development and validation. J. Chronic Dis..

[12] Jang S (2010). Medical service utilization with osteoporosis. Endocrinol. Metab..

[13] Malter, A.D., McNeney, B., Loeser, J.D., Deyo, R.A. 5-year reoperation rates after different types of lumbar spine surgery. *Spine (Phila Pa. 1976).* **23**, 814–820 (1998). 10.1097/00007632-199804010-00015 9563113

[14] Martin BI (2012). Repeat surgery after lumbar decompression for herniated disc: the quality implications of hospital and surgeon variation. Spine J..

[15] Heindel P (2016). Reoperation rates following single-level discectomy. Global Spine J..

[16] Davis RA (1994). A long-term outcome analysis of 984 surgically treated herniated lumbar discs. J. Neurosurg..

[17] Vik A, Zwart JA, Hulleberg G, Nygaard Ø (2001). Eight year outcome after surgery for lumbar disc herniation: a comparison of reoperated and not reoperated patients. Acta Neurochir. (Wien).

[18] Kim, C., et al. The long-term reoperation rate following surgery for lumbar herniated intervertebral disc disease: a nationwide sample cohort study with a 10-year follow-up. *Spine (Phila Pa. 1976).* **44**, 1382–1389 (2019). 10.1097/BRS.0000000000003065 30973508

[19] Jung, J., et al. The long-term reoperation rate following surgery for lumbar stenosis: a nationwide sample cohort study with a 10-year follow-up. *Spine (Phila Pa. 1976).* **45**, 1277–1284 (2020). 10.1097/BRS.0000000000003515 32355142

[20] Bressler, H.B., Keyes, W.J., Rochon, P.A., Badley, E. The prevalence of low back pain in the elderly: a systematic review of the literature. *Spine (Phila Pa. 1976).* **24**, 1813–1819 (1999). 10.1097/00007632-199909010-00011 10488512

[21] Jönsson, B., Annertz, M., Sjöberg, C., Strömqvist, B. A prospective and consecutive study of surgically treated lumbar spinal stenosis: Part II: five-year follow-up by an independent observer. *Spine (Phila Pa. 1976).* **22**, 2938–2944 (1997). 10.1097/00007632-199712150-00017 9431630

[22] Andersen SB, Smith EC, Støttrup C, Carreon LY, Andersen MO (2018). Smoking is an independent risk factor of reoperation due to recurrent lumbar disc herniation. Global Spine J..

[23] Passias PG (2018). Alcoholism as a predictor for pseudarthrosis in primary spine fusion: An analysis of risk factors and 30-day outcomes for 52,402 patients from 2005 to 2013. J. Orthop..

[24] Elsamadicy AA (2017). Impact of alcohol use on 30-day complication and readmission rates after elective spinal fusion (≥2 levels) for adult spine deformity: a single institutional study of 1010 patients. J. Spine Surg. (Hong Kong).

[25] Fritzell P, Knutsson B, Sanden B, Strömqvist B, Hägg O (2015). Recurrent versus primary lumbar disc herniation surgery: patient-reported outcomes in the Swedish spine register Swespine. Clin. Orthop. Relat. Res..

